# Association between life’s essential 8 and colorectal cancer: A population-based study

**DOI:** 10.1097/MD.0000000000043261

**Published:** 2025-07-04

**Authors:** Xiaohan Ma, Xiaofei Zhang, Fasheng Wu, Sheng Chen, Jing Peng, Xuan Lan, Xinyue Zhang, Encun Hou

**Affiliations:** a Graduate School, Guangxi University of Chinese Medicine, Nanning, Guangxi, China; b Ruikang Hospital, Guangxi University of Chinese Medicine, Nanning, Guangxi, China.

**Keywords:** colorectal cancer, life’s essential 8, risk factors, the National Health and Nutrition Examination Survey

## Abstract

Colorectal cancer (CRC) is closely linked to cardiovascular disease. This study investigates the relationship between life’s essential 8 (LE8), a recent cardiovascular health metric, and CRC prevalence in US adults. Data from the 2011 to 2018 National Health and Nutrition Examination Survey were used in this study. LE8 was classified into high, moderate, and low levels. The association between LE8 and CRC was assessed using weighted logistic regression, restricted cubic splines, and subgroup analyses. After adjusting for potential confounders, higher LE8 scores were linked to a lower prevalence of CRC. Individuals with the highest LE8 scores had an 81% reduced risk of CRC compared to those with the lowest scores (odds ratio = 0.19; 95% confidence interval: 0.04–0.88, *P* = .03). Restricted cubic splines analyses revealed a negative correlation between CRC risk and LE8 scores, showing a linear decline as LE8 scores increased (*P* for non-linearity > .05). LE8 was inversely associated with CRC prevalence in a linear manner. Encouraging adherence to optimal cardiovascular health may help reduce CRC burden.

## 1. Introduction

Colorectal cancer (CRC) claims nearly 900,000 lives each year, positioning it as the fourth deadliest cancer globally. This alarming statistic highlights the critical need for enhanced methods in both preventing and detecting the disease early.^[1]^ While significant strides have been made in identifying various risk factors linked to CRC development, these preliminary findings point to an even greater need for intensive research. Deeper investigations into the biological and environmental mechanisms driving CRC will not only clarify its etiology but also pave the way for innovative prevention strategies and more effective early detection methods.^[2–4]^ Moreover, the association between CRC and prevalent systemic diseases such as hypertension, metabolic syndrome, diabetes mellitus, cardiovascular disease (CVD), and chronic kidney disease further illustrates its broad impact on public health.^[5–7]^ These links suggest potential shared risk factors or pathophysiological pathways, which could inform new preventative and therapeutic approaches. Understanding these connections in greater depth could lead to a holistic approach in managing individuals at risk, encompassing both CRC and these commonly associated conditions. This integrative perspective could be pivotal in designing public health policies and healthcare interventions that effectively reduce the incidence and improve the prognosis of CRC, ultimately leading to better health outcomes and decreased mortality.

In 2022, the American Heart Association introduced an advanced enhancement to its scoring system for assessing cardiovascular health, known as the “life’s essential 8” (LE8). This revised scoring framework now includes a comprehensive range of health indicators that more holistically evaluate an individual’s health profile. The components of this system are diet, physical activity, exposure to nicotine, sleep quality, body mass index (BMI), blood lipid levels, blood glucose, and blood pressure. Each of these factors is crucial in determining overall cardiovascular health and now contributes to a cumulative score in the LE8 system.^[8]^ Notably, the scoring is structured such that higher values are indicative of better health status and consequently a lower risk of developing CVD. This inverse relationship between the LE8 scores and cardiovascular risk offers a quantifiable means of evaluating an individual’s health and potentially guiding lifestyle adjustments and medical interventions.

While the LE8 system has been extensively used to assess and monitor cardiovascular health, its application in studying other significant health conditions, particularly CRC, has not yet been pursued. Previous research has robustly established the LE8 as a reliable indicator of cardiovascular health, but its potential utility in the context of CRC risk assessment remains largely untapped. Given the system’s comprehensive nature, which covers various aspects of health that could influence cancer risk, such as diet and physical activity, it stands to reason that LE8 could provide valuable insights into the relationship between lifestyle factors and the likelihood of developing CRC. We aim to address this significant gap in the existing literature, hypothesizing that the holistic health measures captured by the LE8 could correlate with CRC risks similarly to their known associations with cardiovascular health. By extending the use of LE8 beyond its traditional scope, this research could pave the way for more integrated approaches to disease prevention, encompassing a wider range of health outcomes and potentially offering novel insights into disease interconnections and shared risk factors.

## 2. Methods

### 2.1. Data source

The National Health and Nutrition Examination Survey (NHANES), conducted by the National Center for Health Statistics, is a comprehensive study that evaluates the health and nutritional status of the U.S. population. All participants provided written informed consent, and the study was reviewed and approved by the National Center for Health Statistics Ethics Review Committee.^[9]^

### 2.2. Study population

**Figure 1.**
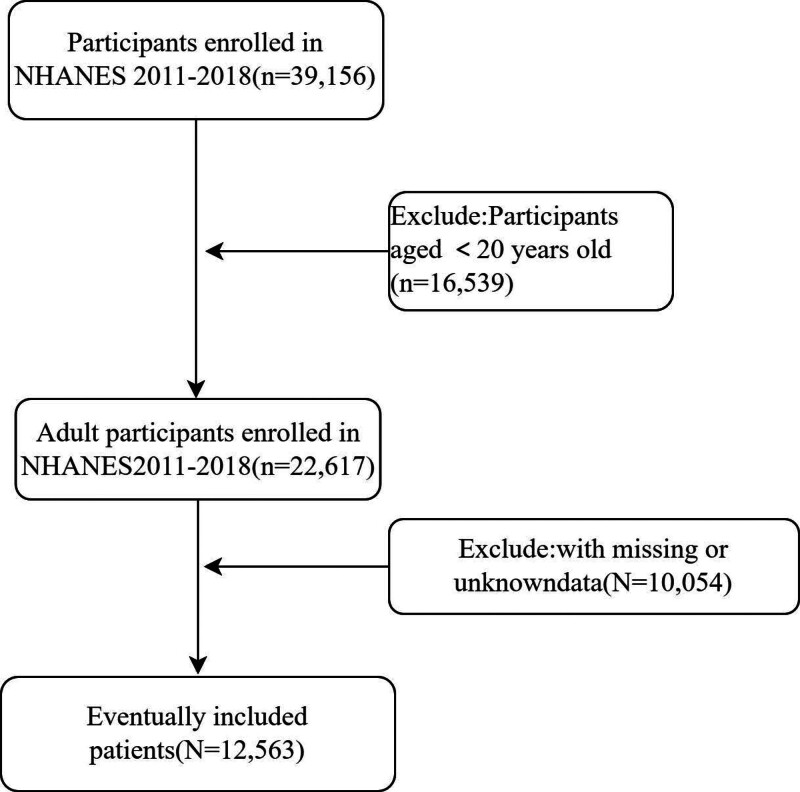
Flowchart of the sample selection from NHANES 2011 to 2018. NHANES = National Health and Nutrition Examination Survey.

As illustrated in Figure 1 of our research documentation, this study specifically targeted individuals aged 20 years and older. Participants were drawn from a comprehensive pool of respondents who took part in NHANES over a span of 8 years, from 2011 to 2018. The initial dataset comprised a substantial number of 39,156 participants, providing a broad basis for analyzing the health metrics in question. However, in the preliminary phase of data processing and preparation, a significant proportion of these records were found to be incomplete and thus not suitable for the rigorous analytical methods required in this study. Specifically, 26,593 participants were excluded from further analysis due to missing information that was critical for the reliable application of the LE8 scoring system. Such data gaps primarily involved one or more of the 8 essential health metrics that the LE8 scoring system evaluates. After this necessary exclusion of incomplete datasets, the study proceeded with a robust final sample of 12,563 participants. This filtered group formed the basis of our investigation, ensuring that the insights and findings derived would be based on comprehensive and accurately measured data. This approach not only strengthens the validity of our study but also enhances the reliability of our conclusions regarding the relationship between LE8 scores and health outcomes, particularly in assessing the risk factors associated with colorectal cancer in the context of diverse health behaviors and biometric indicators.

### 2.3. Exposure

The LE8 scoring algorithm, as designed by the American Heart Association, incorporates a comprehensive set of metrics that are split into 2 main categories: behavioral metrics and biometric health indicators. The behavioral metrics include diet, physical activity, nicotine exposure, and sleep duration, each reflecting lifestyle choices that significantly impact overall health. The biometric health indicators consist of BMI, non-high-density lipoprotein cholesterol, blood glucose, and blood pressure, which are critical clinical measures that provide a snapshot of an individual’s physiological health status. Together, these 8 components form the foundation of the LE8 scoring system, enabling a holistic assessment of cardiovascular health.^[10]^

In the context of this study, the LE8 scores of participants are categorized into 3 distinct ranges that reflect varying levels of health. Individuals with scores between 80 to 100 are categorized as having high scores, indicating optimal health behaviors and biometric readings, suggesting that they adhere closely to recommended health guidelines and are at a lower risk of developing cardiovascular conditions. Participants with scores ranging from 50 to 79 are classified as having moderate scores. This group exhibits a mix of adequate and potentially risky health behaviors and biometric results, pointing to a moderate risk level that may necessitate targeted health interventions. Lastly, scores from 0 to 49 are considered low, denoting poor adherence to health-promoting behaviors and unfavorable biometric readings, which correlate with a higher risk of cardiovascular issues and other health complications.

The diet metric was assessed using the Healthy Eating Index 2015.^[11]^ Questionnaire data included physical activity, nicotine exposure, sleep patterns, and diabetes status. Laboratory data provided information on blood lipids and glucose, while blood pressure, height, and weight were measured at the mobile examination center. BMI was calculated by dividing weight in kilograms by the square of height in meters.

### 2.4. Outcome

CRC-associated questions included: “Have you ever been told by a doctor or health professional that you had CRC?.” The participants who answer “yes” to the question defined patients with CRC. After removing missing values and including variables, 12,563 people overall (72 of whom had CRC) completed the NHANES 2011 to 2018 questionnaire.

### 2.5. Covariables

Variables included age, sex, marital status, ethnicity (categorized into Mexican American, non-Hispanic Black, non-Hispanic White, other), education level (divided into “less than high school and “high school or above.”)^[12]^ Ratio of family income to poverty (PIR) was divided into ≤ 1, 1 to 3, and ≥ 3 based on household income to PIR threshold ratio.^[13]^ An affirmative answer to any of the following statements was regarded as an indication of CVD: being informed of having congestive heart failure, coronary heart disease, angina/angina pectoris, a heart attack, or a stroke.^[14]^

### 2.6. Statistical analysis

Data were analyzed according to NHANES analytical guidelines and recommended survey weights. Demographic characteristics related to CRC status were assessed using chi-square and *t* tests. Categorical variables are presented as weighted percentages, while continuous variables are presented as mean ± standard error .

The independent association between LE8 and its components with CRC was investigated through survey-weighted multivariable logistic regressions, adjusting for potential confounders. The association between LE8 and CRC risk was further explored using restricted cubic spline analysis.

To examine the association between LE8 and CRC across different populations, we conducted stratified analyses based on age, sex, ethnicity, and education levels. The significance of interactions was assessed using *P* values for the interaction coefficients between LE8 and subgroup populations.

All statistical analyses were conducted using R software. Statistical tests were two-sided, and significance was assumed at *P* < .05.

## 3. Results

### 3.1. Baseline characteristics of respondents

Table 1 outlines the baseline characteristics of the 12,563 participants. Participants with and without CRC significantly differed in several factors, including age, LE8 score, physical activity, blood glucose, blood pressure, ethnicity, CVD, and lipid levels (all *P* < .05). These differences highlight key variables potentially associated with CRC status.

**Table 1:** Baseline characteristics of respondents.

Variable	Total	Non-CRC	CRC	*P*-value
HEI-2015 diet score, mean ± SE	38.9 ± 0.6	38.8 ± 0.6	41.9 ± 4.8	.5
Physical activity score, mean ± SE	75.2 ± 0.7	75.3 ± 0.7	53.2 ± 6.7	.002
Nicotine exposure score, mean ± SE	73.0 ± 0.6	73.1 ± 0.6	68.3 ± 5.8	.4
Sleep health score, mean ± SE	83.9 ± 0.4	83.9 ± 0.4	82.7 ± 4.7	.8
BMI score, mean ± SE	59.0 ± 0.6	59.0 ± 0.6	52.4 ± 5.6	.2
Blood lipids score, mean ± SE	66.3 ± 0.5	66.3 ± 0.5	56.7 ± 4.2	.03
Blood glucose score, mean ± SE	85.9 ± 0.3	85.9 ± 0.3	73.8 ± 4.6	.01
Blood pressure score, mean ± SE	70.2 ± 0.5	70.3 ± 0.5	50.6 ± 4.4	<.0001
LE8 (N, weighted %)				.01
Low	1494 (9.4)	1482 (9.4)	12 (19.4)	
Moderate	8260 (64.4)	8204 (64.4)	56 (73.2)	
High	2809 (26.1)	2805 (26.2)	4 (7.4)	
Age (N, weighted %)				<.0001
<65	10,122 (84.3)	10,100 (84.5)	22 (38.7)	
≥65	2441 (15.7)	2391 (15.5)	50 (61.3)	
Sex (N, weighted %)				.6
Male	6097 (48.9)	6059 (48.9)	38 (52.6)	
Female	6466 (51.1)	6432 (51.1)	34 (47.4)	
Ethnicity (N, weighted %)				.002
Non-Hispanic White	4873 (66.9)	4832 (66.8)	41 (82.9)	
Other	3193 (14.2)	3181 (14.2)	12 (6.4)	
Non-Hispanic Black	2820 (10.7)	2807 (10.7)	13 (7.1)	
Mexican American	1677 (8.3)	1671 (8.3)	6 (3.6)	
PIR (N, weighted %)				1
≤1	2681 (14.1)	2667 (14.2)	14 (13.9)	
1–3	5200 (35.7)	5168 (35.7)	32 (37.2)	
≥3	4682 (50.2)	4656 (50.2)	26 (48.8)	
Education (N, weighted %)				.5
≤High school	5201 (35.1)	5167 (35.1)	34 (31.1)	
>High school	7362 (64.9)	7324 (64.9)	38 (68.9)	
Marital status (N, weighted %)				.6
Single or separated	5069 (36.1)	5035 (36.0)	34 (39.5)	
Coupled	7494 (63.9)	7456 (64.0)	38 (60.5)	
CVD (N, weighted %)				<.001
No	11,370 (92.5)	11,313 (92.6)	57 (79.0)	
Yes	1193 (7.5)	1178 (7.4)	15 (21.0)	

Data are presented as weighted mean ± SE or weighted frequencies (weighted percentages).

BMI = body mass index, CVD = cardiovascular disease, HEI = indicates Healthy Eating Index, LE8 = life’s essential 8, PIR = poverty to income ratio, SE = standard error.

### 3.2. The association between LE8 scores and CRC

The results show a reduced CRC risk with increasing LE8 scores (Table 2). In the initial crude model, LE8 scores as a continuous variable were significantly associated with lower CRC risk. This association remained significant after adjusting for various confounders. When LE8 score was treated as a categorical variable in Model 2, participants with the highest LE8 score had a significantly lower risk of CRC compared to those with the lowest score, with an odds ratio of 0.19 (95% confidence interval: 0.04–0.88).

**Table 2:** Multiple logistic regression models of LE8 scores with CRC.

	Crude model		Model 1		Model 2	
	OR (95% CI)	*P*	OR (95% CI)	*P*	OR (95% CI)	*P*
LE8 (continuous)	0.96 (0.95, 0.98)	<.0001	0.96 (0.94, 0.98)	<.001	0.96 (0.94, 0.98)	.002
LE8 (multi-category)						
Low	Ref.		Ref.		Ref.	
Moderate	0.55 (0.22, 1.38)	.20	0.58 (0.20, 1.62)	.29	0.58 (0.20, 1.71)	.32
High	0.14 (0.03, 0.55)	.01	0.19 (0.04, 0.85)	.03	0.19 (0.04, 0.88)	.03
*P* for trend		.001		.02		.03

Crude model: no adjustment for any potential influence factors.

Model 1: adjusted for age, sex, ethnicity.

Model 2: adjusted for age, sex, ethnicity, education level, PIR, marital status, CVD.

BMI = body mass index, CI = confidence interval, CRC = colorectal cancer, CVD = cardiovascular disease, LE8 = life’s essential 8, OR = odds ratio, PIR = poverty to income ratio.

**Figure 2.**
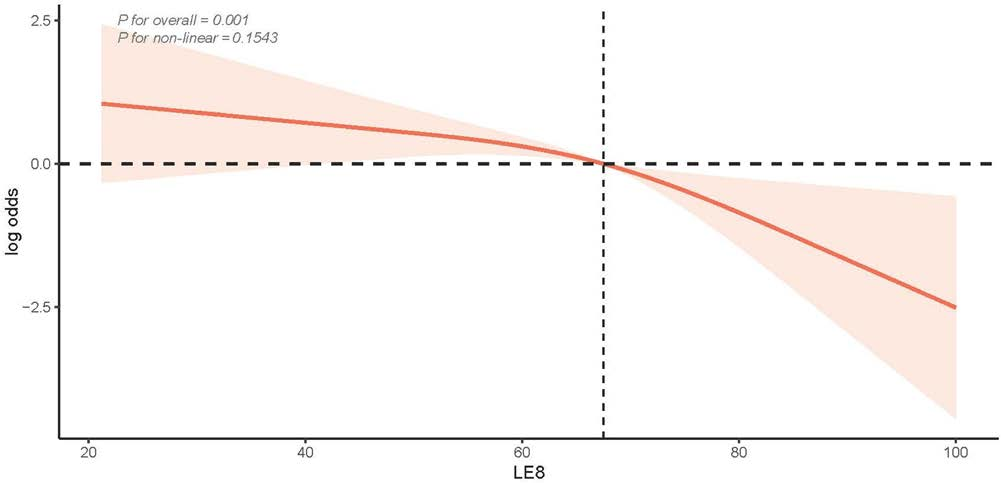
Dose–response relationships between LE8 scores and CRC prevalence. CRC = colorectal cancer, LE8 = life’s essential 8.

According to the restricted cubic spline analysis, the LE8 score showed an approximately linear relationship with the risk of developing CRC (overall *P* < .05, nonlinear *P* > .05; Fig. 2). This finding suggests that as the LE8 score increases, the risk of CRC decreases in a linear fashion. The analysis supports the idea that better cardiovascular health, as reflected by higher LE8 scores, is associated with a progressively lower risk of developing CRC. This linear trend indicates that improvements in cardiovascular health could potentially reduce the risk of CRC, reinforcing the importance of maintaining optimal cardiovascular health levels.

### 3.3. Subgroup analysis

**Figure 3.**
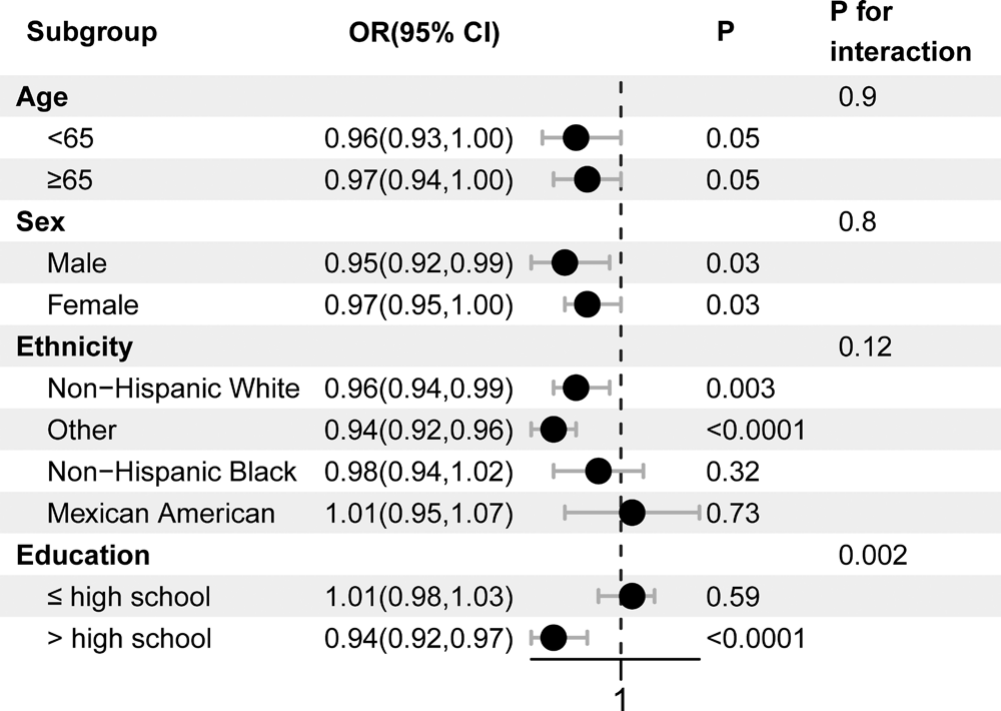
Subgroup analysis for the association between LE8 scores and CRC. Adjusted for PIR, marital status, and CVD (except for grouping covariates). CRC = colorectal cancer, CVD = cardiovascular disease, LE8 = life’s essential 8, PIR = poverty to income ratio.

A stratified analysis was conducted to examine the relationship between LE8 score and CRC risk across various demographic and health-related factors, as shown in Figure 3. To better understand the impact of potential confounders on the association between LE8 scores and CRC, participants were grouped into subgroups based on age, sex, race, and education level. Statistically significant associations were found for gender, non-Hispanic whites, other racial groups, and individuals with education levels beyond high school. Additionally, a significant interaction between LE8 scores and education level was observed (*P*-value for interaction < .05), suggesting that education level may influence the strength of the association between LE8 scores and CRC risk.

## 4. Discussion

The findings of this study distinctly highlight a negative correlation between the LE8 score and the likelihood of CRC. This means that higher LE8 scores, which indicate better adherence to the 8 key health metrics assessed (including factors like diet, exercise, and vital health measures) are consistently associated with a reduced risk of developing CRC. The data vividly illustrates that individuals maintaining a higher standard of cardiovascular health, as quantified by the LE8 scores, are less likely to be diagnosed with CRC.

Interestingly, the study also highlighted that the protective effect of higher LE8 scores against CRC differs according to educational attainment. Specifically, individuals with education beyond high school were found to have a lower likelihood of developing CRC compared to those with a high school education or less. Previous studies in Sweden and the US have shown that CRC incidence rates are significantly higher in individuals with lower secondary or high school education compared to those with university or higher education.^[15,16]^ This may be due to the lower risk of CVD with higher levels of education, which reduces the risk of colorectal cancer.

Studies have consistently shown a significant link between CVD and an increased risk of cancer development.^[17]^ Furthermore, additional research indicates that individuals with lower levels of education tend to have a higher risk of developing CVD.^[18]^ Education may influence health outcomes through several pathways.^[19]^ One of the most notable factors is the vocational opportunities that education provides, which are closely tied to access to healthcare in the US. Contemporary NHANES data reveal a cross-sectional relationship between educational attainment and access to both general and specialty care among individuals with CVD.^[20]^ Educational attainment is closely linked to health literacy and health-related behaviors, both of which may influence CVD risk. Higher levels of education are associated with better understanding of health information and more proactive health behaviors, which can help reduce cardiovascular risk.^[21]^ Research has consistently shown a significant relationship between educational attainment and various cardiovascular risk factors, such as smoking, cholesterol levels, physical activity, and the achievement of ideal cardiovascular health.^[22,23]^ These studies suggest that education plays a key role in shaping behaviors that contribute to cardiovascular health and disease prevention.

Given these findings, it is evident that further investigation is necessary to thoroughly understand the factors underlying the observed association between education levels and CRC risk. This exploration should consider how educational attainment influences health literacy, access to healthcare resources, and engagement in preventive health behaviors, which may collectively or individually contribute to the variations in CRC incidence. Such research could provide deeper insights into the mechanisms through which education and lifestyle choices impact CRC risk and could inform targeted interventions aimed at reducing health disparities.

The utility and significance of LE8 as a metric for evaluating cardiovascular health are widely acknowledged. Higher LE8 scores have been linked to decreased risks of all-cause and CVD-specific mortality. Conversely, individuals with lower LE8 scores face elevated risks of major adverse cardiovascular events and specific cardiovascular outcomes.^[24,25]^ The established correlation between LE8 and CVD is indisputable. Furthermore, LE8 has been associated with chronic kidney disease and nonalcoholic fatty liver disease.^[26,27]^ Despite these findings, investigations into the relationship between LE8 and CRC have not been documented. Thus, there is ample justification to explore the potential link between LE8 and CRC. In our study, we have diligently pursued this exploration and provided comprehensive elucidation of their relationship. The potential reasons for their association are summarized below.

This finding aligns with previous studies demonstrating a higher risk of CRC occurrence with increased fat intake, contrasting with the protective effects of a diet rich in fruits and vegetables.^[28]^ Diabetes, regardless of BMI, physical activity, or smoking status, is identified as a risk factor for colorectal cancer.^[29,30]^ The underlying pathophysiological mechanism involves elevated serum levels of insulin and IGF-1, which promote the proliferation of colorectal epithelial cells.^[31]^

The results of this study underscore the intricate relationship between the components of the LE8 score and the risk of CRC. Given that the LE8 score encompasses a broad spectrum of health indicators (ranging from diet and physical activity to crucial clinical metrics like blood glucose and cholesterol levels) it is evident that these factors are not only essential for cardiovascular health but also significantly impact CRC risk. Therefore, employing the LE8 score as a tool for evaluating the risk of CRC appears to be a logical and practical approach. Moreover, this relationship serves as a powerful reminder of the importance of maintaining a healthy lifestyle, especially for individuals who are at an increased risk of developing CRC. Adopting and adhering to health-promoting behaviors that directly influence the LE8 score can potentially mitigate this risk. These behaviors include maintaining a balanced diet, engaging in regular physical activity, managing body weight, and avoiding harmful exposures such as nicotine. Additionally, the use of the LE8 score as a holistic measure encourages a more comprehensive approach to health management, emphasizing prevention across a spectrum of diseases, including CRC. It prompts healthcare providers and individuals alike to consider not only the direct impacts of their health choices but also the broader implications for disease prevention. This perspective is crucial for those at higher CRC risk, highlighting the need for proactive engagement in health-enhancing practices that could significantly alter disease outcomes.

The data for this study were derived from the NHANES database, which is renowned for its comprehensive scope and robustness, offering several distinct advantages. The NHANES database is highly representative of the broader American population, thanks to its methodical sampling strategy, ensuring that the findings from studies using this data can be broadly applicable within the United States. Additionally, the database is publicly accessible, making it a valuable resource for researchers looking to explore various health-related questions. Furthermore, the user-friendly nature of the NHANES system facilitates its use among researchers, promoting a wider dissemination of knowledge and encouraging a diverse range of scientific inquiries.

Despite its strengths, the study has several limitations that must be acknowledged. A primary limitation is the cross-sectional design of the NHANES data, which prevents the establishment of causality. While this design can identify associations between variables, it cannot definitively determine whether 1 variable causes another. This is a critical distinction that limits the ability to draw firm conclusions about the effect of the LE8 score on CRC risk from this data alone. Another limitation arises from the demographic scope of the NHANES database, which includes only American participants. Consequently, the findings may not be directly applicable to populations in other countries, whose environmental, genetic, and lifestyle factors might differ significantly from those of the American population. This limitation restricts the generalizability of the results, necessitating caution when extrapolating these findings to other groups. Lastly, the results of this study, while informative, cannot lead directly to definitive conclusions. The cross-sectional nature of the data means that further research, ideally through prospective longitudinal studies, is necessary to validate and expand upon these findings. Longitudinal studies would allow researchers to track changes over time, providing a clearer picture of how the LE8 score impacts CRC risk across different stages of life and under varying conditions, thus offering a more robust framework for understanding the dynamics at play.

## 5. Conclusions

In this retrospective cohort study, our observations strongly suggest that maintaining a healthy lifestyle, as evidenced by higher LE8 scores, is potentially associated with a reduced prevalence of CRC.

## Author contributions

**Data curation:** Xuan Lan.

**Formal analysis:** Xiaofei Zhang, Fasheng Wu.

**Funding acquisition:** Xiaofei Zhang, Fasheng Wu, Encun Hou.

**Investigation:** Xiaofei Zhang, Fasheng Wu, Xinyue Zhang.

**Methodology:** Xiaofei Zhang, Fasheng Wu, Jing Peng, Xuan Lan, Xinyue Zhang.

**Resources:** Xiaohan Ma, Jing Peng, Xuan Lan.

**Software:** Xiaohan Ma, Xiaofei Zhang, Sheng Chen, Jing Peng, Xinyue Zhang, Encun Hou.

**Supervision:** Xiaofei Zhang, Fasheng Wu, Encun Hou.

**Validation:** Fasheng Wu.

**Visualization:** Xiaofei Zhang.

**Writing – original draft:** Xiaohan Ma, Xiaofei Zhang, Fasheng Wu, Sheng Chen.

**Writing – review & editing:** Xiaohan Ma, Xiaofei Zhang, Fasheng Wu, Sheng Chen.

## References

[1] Bray F Ferlay J Soerjomataram I Siegel RL Torre LA Jemal A. Global cancer statistics 2018: GLOBOCAN estimates of incidence and mortality worldwide for 36 cancers in 185 countries. CA Cancer J Clin. 2018;68:394–424. 30207593 10.3322/caac.21492

[2] Patel SG Karlitz JJ Yen T Lieu CH Boland CR. The rising tide of early-onset colorectal cancer: a comprehensive review of epidemiology, clinical features, biology, risk factors, prevention, and early detection. Lancet Gastroenterol Hepatol. 2022;7:262–74. 35090605 10.1016/S2468-1253(21)00426-X

[3] Hull MA Rees CJ Sharp L Koo S. A risk-stratified approach to colorectal cancer prevention and diagnosis. Nat Rev Gastroenterol Hepatol. 2020;17:773–80. 33067592 10.1038/s41575-020-00368-3 PMC7562765

[4] Rickels MR Robertson RP. Pancreatic islet transplantation in humans: recent progress and future directions. Endocr Rev. 2019;40:631–68. 30541144 10.1210/er.2018-00154 PMC6424003

[5] Chen Y Kong W Liu M. Metabolic syndrome and risk of colorectal cancer: a Mendelian randomization study. Heliyon. 2024;10:e23872. 38223733 10.1016/j.heliyon.2023.e23872 PMC10784169

[6] O’Sullivan DE Sutherland RL Town S. Risk factors for early-onset colorectal cancer: a systematic review and meta-analysis. Clin Gastroenterol Hepatol. 2022;20:1229–40.e5. 33524598 10.1016/j.cgh.2021.01.037

[7] Xuan K Zhao T Sun C. The association between hypertension and colorectal cancer: a meta-analysis of observational studies. Eur J Cancer Prev. 2021;30:84–96. 32039929 10.1097/CEJ.0000000000000578

[8] Shetty NS Parcha V Patel N. AHA Life’s essential 8 and ideal cardiovascular health among young adults. Am J Prev Cardiol. 2023;13:100452. 36636126 10.1016/j.ajpc.2022.100452 PMC9830108

[9] von Elm E Altman DG Egger M. The Strengthening the Reporting of Observational Studies in Epidemiology (STROBE) Statement: guidelines for reporting observational studies. Int J Surg. 2014;12:1495–9. 25046131 10.1016/j.ijsu.2014.07.013

[10] Lloyd-Jones DM Allen NB Anderson CAM. Life’s Essential 8: updating and enhancing the American Heart Association’s construct of cardiovascular health: a presidential advisory from the American Heart Association. Circulation. 2022;146:e18–43. 35766027 10.1161/CIR.0000000000001078 PMC10503546

[11] Krebs-Smith SM Pannucci TE Subar AF. Update of the healthy eating index: HEI-2015. J Acad Nutr Diet. 2018;118:1591–602. 30146071 10.1016/j.jand.2018.05.021 PMC6719291

[12] McQuillan J Andersen JA Berdahl TA Willett J. Associations of rheumatoid arthritis and depressive symptoms over time: are there differences by education, race/ethnicity, and gender? Arthritis Care Res (Hoboken). 2022;74:2050–8. 34121353 10.1002/acr.24730

[13] Okosun IS Annor FB Seale JP Eriksen MP. Abdominal adiposity and family income-to-poverty ratio in American women. Obes Res Clin Pract. 2014;8:e201–298. 10.1016/j.orcp.2012.12.002 24847661

[14] Scinicariello F Buser MC Feroe AG Attanasio R. Antimony and sleep-related disorders: NHANES 2005-2008. Environ Res. 2017;156:247–52. 28363141 10.1016/j.envres.2017.03.036 PMC5685481

[15] Brooke HL Talbäck M Martling A Feychting M Ljung R. Socioeconomic position and incidence of colorectal cancer in the Swedish population. Cancer Epidemiol. 2016;40:188–95. 26773279 10.1016/j.canep.2016.01.004

[16] Doubeni CA Laiyemo AO Major JM. Socioeconomic status and the risk of colorectal cancer: an analysis of more than a half million adults in the National Institutes of Health-AARP Diet and Health Study. Cancer. 2012;118:3636–44. 22898918 10.1002/cncr.26677 PMC3422782

[17] Meijers WC Maglione M Bakker SJL. Heart failure stimulates tumor growth by circulating factors. Circulation. 2018;138:678–91. 29459363 10.1161/CIRCULATIONAHA.117.030816

[18] Johnson AE Herbert BM Stokes N Brooks MM Needham BL Magnani JW. Educational attainment, race, and ethnicity as predictors for ideal cardiovascular health: from the National Health and Nutrition Examination Survey. J Am Heart Assoc. 2022;11:e023438. 34984911 10.1161/JAHA.121.023438 PMC9238535

[19] Braveman P Egerter S Williams DR. The social determinants of health: coming of age. Annu Rev Public Health. 2011;32:381–98. 21091195 10.1146/annurev-publhealth-031210-101218

[20] Marmot M Bell R. Challenging health inequalities--implications for the workplace. Occup Med (Lond). 2010;60:162–4. 20423942 10.1093/occmed/kqq008

[21] Magnani JW Mujahid MS Aronow HD. Health literacy and cardiovascular disease: fundamental relevance to primary and secondary prevention: a scientific statement from the American Heart Association. Circulation. 2018;138:e48–74. 29866648 10.1161/CIR.0000000000000579 PMC6380187

[22] Hamad R Nguyen TT Bhattacharya J Glymour MM Rehkopf DH. Educational attainment and cardiovascular disease in the United States: a quasi-experimental instrumental variables analysis. PLoS Med. 2019;16:e1002834. 31237869 10.1371/journal.pmed.1002834 PMC6592509

[23] Mackenbach JP Cavelaars AE Kunst AE Groenhof F. Socioeconomic inequalities in cardiovascular disease mortality; an international study. Eur Heart J. 2000;21:1141–51. 10924297 10.1053/euhj.1999.1990

[24] Sun J Li Y Zhao M. Association of the American Heart Association’s new “Life’s Essential 8” with all-cause and cardiovascular disease-specific mortality: prospective cohort study. BMC Med. 2023;21:116. 36978123 10.1186/s12916-023-02824-8 PMC10053736

[25] Petermann-Rocha F Deo S Celis-Morales C. An opportunity for prevention: associations between the Life’s Essential 8 score and cardiovascular incidence using prospective data from UK Biobank. Curr Probl Cardiol. 2023;48:101540. 36528209 10.1016/j.cpcardiol.2022.101540

[26] Chen H Tang H Huang J Luo N Zhang X Wang X. Life’s Essential 8 and mortality in US adults with chronic kidney disease. Am J Nephrol. 2023;54:516–27. 37591229 10.1159/000533257

[27] Wang L Yi J Guo X Ren X. Associations between life’s essential 8 and non-alcoholic fatty liver disease among US adults. J Transl Med. 2022;20:616. 36564799 10.1186/s12967-022-03839-0 PMC9789599

[28] Schulz MD Atay C Heringer J. High-fat-diet-mediated dysbiosis promotes intestinal carcinogenesis independently of obesity. Nature. 2014;514:508–12. 25174708 10.1038/nature13398 PMC4233209

[29] Yuhara H Steinmaus C Cohen SE Corley DA Tei Y Buffler PA. Is diabetes mellitus an independent risk factor for colon cancer and rectal cancer? Am J Gastroenterol. 2011;106:1911–21; quiz 1922. 21912438 10.1038/ajg.2011.301 PMC3741453

[30] Kim DS Scherer PE. Obesity, diabetes, and increased cancer progression. Diabetes Metab J. 2021;45:799–812. 34847640 10.4093/dmj.2021.0077 PMC8640143

[31] Murphy N Song M Papadimitriou N. Associations between glycemic traits and colorectal cancer: a Mendelian randomization analysis. J Natl Cancer Inst. 2022;114:740–52. 35048991 10.1093/jnci/djac011 PMC9086764

